# Termite Resistance of Thermally-Modified *Dendrocalamus asper* (Schultes f.) Backer ex Heyne

**DOI:** 10.3390/insects3020390

**Published:** 2012-03-27

**Authors:** Ronniel D. Manalo, Carlos M. Garcia

**Affiliations:** 1 Department of Forest Products and Paper Science, College of Forestry and Natural Resources, University of the Philippines Los Baños, Laguna 4031, Philippines; 2 Department of Science and Technology, Forest Products Research and Development Institute, Los Baños, Laguna 4031, Philippines; E-Mail: cmgarcia_losbanos@yahoo.com

**Keywords:** *Dendrocalamusasper*, virgin coconut oil, oil heat treatment, *Microcerotermeslosbañosensis*

## Abstract

The effects of thermal modification on the resistance of *Dendrocalamus asper* against *Microcerotermes losbañosensis* were investigated after exposure to virgin coconut oil at 140–200 °C for 30–120 min. The results showed that heat treatment significantly improved bamboo’s resistance to termites based on mass losses and visual observations. The enhancement was highest at 200 °C. Prolonged treatment had a positive effect on the resistance at lower temperatures only.

## 1. Introduction

Bamboo has been gaining interest worldwide as one of the most important non-timber forest products with the shift in its uses from food, construction materials, handicrafts and furniture to pulp and paper, panel boards and reconstituted panel products. The growth of bamboo-related industries is attributed to advances in processing technology and increased market demand for timber substitutes. However, this has resulted in the fast depletion of bamboo. In addition, bamboo is susceptible to microorganisms (e.g., decay fungi) and xylophaegous boring insects, strongly suggesting the need for proper use and protection to maximize its use.

Several treatment methods have been developed to prolong bamboo’s usefulness using preservatives [1,2,3]. However, increased public concern about the environment has given impetus to the search for more earth-friendly treatment methods. One of these, which has piqued the interest of scientists, is thermal modification using different types of vegetable oils [4,5,6,7]. These oils facilitate fast and uniform heat transfer and provide limited oxygen in the heating vessel [8,9]. Most studies on oil heat treatment of bamboo relate only to physical and mechanical properties, and very few on durability, specifically on decay resistance and none on termites. Research proved that oil heat treatment improved the resistance of bamboo against fungal decay [4,5,6]. However, its efficacy against termites has not been tested, which provided impetus to this study. This paper reports the effects of oil heat treatment on the resistance of *Dendrocalamus asper* against the subterranean termite *Microcerotermes losbañosensis* Oshima (Isoptera: Termitidae).

## 2. Experimental Section

### 2.1. Sample Preparation

Three culms or stems of three-year old *D. asper* were collected from the bambusetum at the Los Baños Experimental Station of the Ecosystem Research and Development Bureau (ERDB), Los Baños, Laguna, Philippines. The culms were cut into approximately two-meter lengths and transported to the laboratory of the Department of Forest Products and Paper Science, College of Forestry and Natural Resources, University of the Philippines Los Baños (UPLB) for sample preparation. 

Nodes were removed from the culms and only culms without any sign of defect, visible mold infection and discoloration were selected for the present study. Culm parts from the 10th to the 15th internodes only were split into 25.4 × 300 mm slats. Average culm thickness was 11.50 mm. All slats were conditioned at 20 ± 2 °C and 65% RH until the moisture content reached 12%.

### 2.2. Thermal Modification

The slat samples were immersed in hot oil using a fabricated electric oil curing apparatus. The apparatus consisted of a stainless steel cylindrical vat (300 mm in diameter and 450 mm in height) heated by electric plates (6,800 watts) equipped with a thermocouple and digital temperature controller. Virgin coconut oil (VCO Grade B) with specific gravity of 0.92, viscosity of 1.5 cps, pH 5.9 and smoke point of 212 °C was supplied by the National Institute of Molecular Biology and Biotechnology, UPLB. The samples were completely submerged in the 60 °C-heated oil. Oil temperature was raised to 140, 160, 180 and 200 °C and maintained for 30, 60 and 120 min. Treatment time started when the oil bath reached the target temperature. The samples were then blotted with paper and allowed to cool for 24 h.

### 2.3. Resistance against Subterranean Termites (FPRDI Laboratory Method)

**Figure 1:**
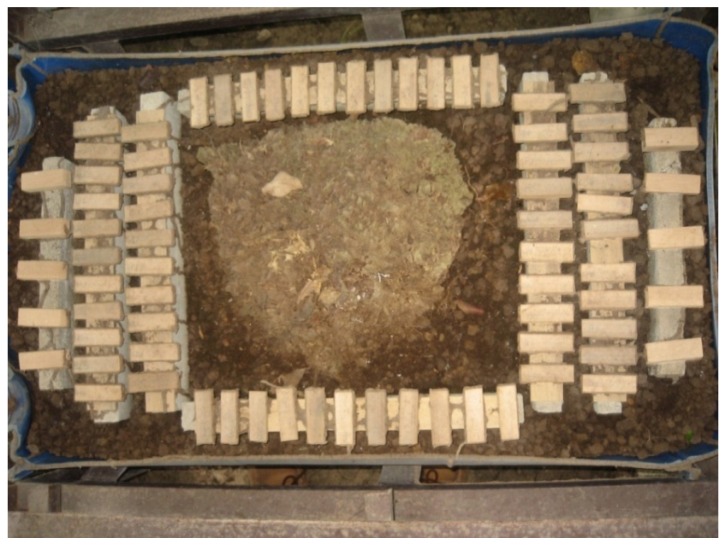
Experimental set-up for the termite resistance test.

Test bamboo samples measuring 20 mm × 60 mm × actual thickness were prepared, 50.8 mm from the ends of the treated and untreated slats. The samples were conditioned for 4 weeks using the specifications described in Section 2.1. A total of five replicates per treatment (including untreated controls) were prepared. A secondary nest of *M. losbañosensis* collected from the UPLB Campus was placed in a halved plastic drum (200 L) with soil. The nest was kept intact for two weeks for termites to feed on the wood slats placed at the far ends of the set-up. After which, the thermally treated and untreated control bamboo samples were placed randomly around the nest with the inner faces in contact with the soil (Figure 1).

**Figure 2:**
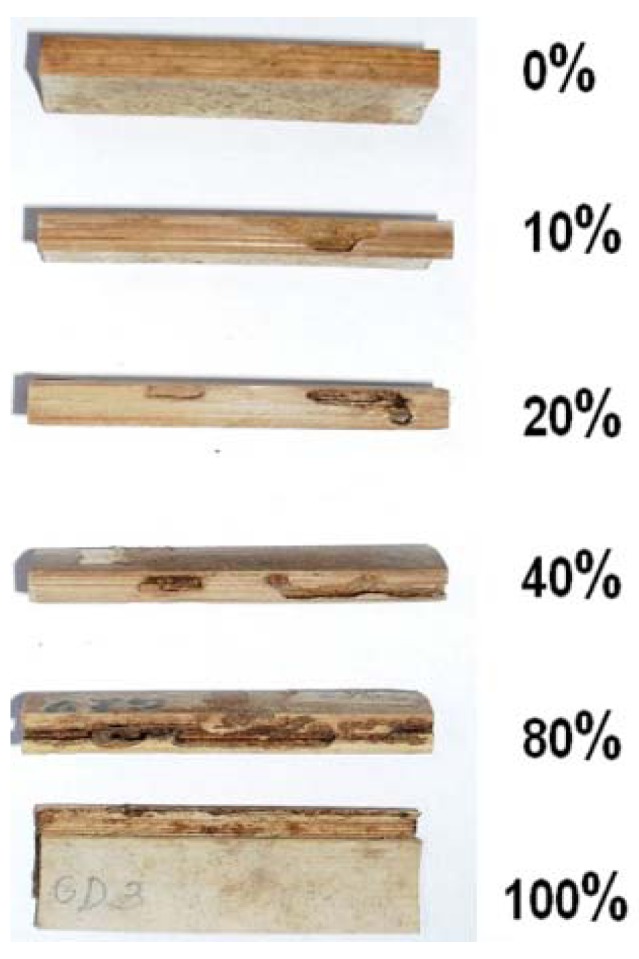
Visual rating of termite attack on bamboo test blocks.

The experimental set-up was inspected every other day for the first 2 weeks, and the tunneling activities of termites were recorded. Weekly observations were done subsequently for 14 weeks when the test was terminated for evaluation. The degree of termite damage on the bamboo samples was visually assessed based on a rating scale of 0 to 100%, which corresponded to increasing damage on bamboo (Figure 2).

In addition to visual assessment, termite damage was gauged from mass loss of each sample, which was calculated from the difference in oven-dried weights before and after the termite test. Data were subjected to an analysis of variance (ANOVA) fitted in 4 × 3 factorial in a completely randomized design (CRD) and means were separated using Tukey’s highly significant difference test (HSD, α = 0.05) [10].

## 3. Results and Discussion

The results showed that thermal modification improved *D. asper*’s resistance against *M. losbañosensis* (Table 1). Average mass losses were 56.74, 50.47, 43.88, and 34.09% for the oil heat treated samples at 140, 160, 180, and 200 °C treatments, respectively, compared to 79.73% for untreated ones. Treatment temperature levels from 140 to 200 °C had a highly significant effect on the mass loss (*p* < 0.001). Exposure of the bamboo samples to higher temperature levels (180 and 200 °C) increased the termite resistance as compared to lower levels. However, effects of holding period from 30 to 120 min on the mass loss were not significant (*p* > 0.348). In addition, heat treatments longer than 30 min seemed to have a positive effect on resistance specifically at lower temperature levels (140 and 160 °C). This, however, was not true for the higher temperature levels (180 and 200 °C). Interactions between temperature and holding period were also not significant (*p* > 0.405). The results showed that temperature has a more dominant role in oil heat treatment.

**Table 1:** Summary of % mass loss after a 16-week exposure of *D. asper* to *M. losbañosensis*.

Treatment Temperature (°C)	Holding Period (min)		
	30	60	120
140	62.42 ±13.33 ^a^	53.02 ± 7.77 ^ab^	54.76 ± 7.91 ^ab^
160	59.04 ± 8.47 ^a^	46.11 ± 13.30 ^ab^	46.25 ± 14.05 ^ab^
180	40.95 ± 9.04 ^ab^	46.61 ± 5.76 ^ab^	44.07 ± 6.05 ^ab^
200	33.70 ± 12.89 ^b^	34.51 ± 9.22 ^b^	34.06 ± 10.12 ^b^
control	79.73 ±3.32		

Means having the same letter/s in each column are not significantly different at α = 0.05.

Visual observations supported the results of the mass losses. The mean damage ratings regardless of holding period were 100.00, 92.33, 83.67, 80.00 and 54.00% for the untreated and the 140, 160, 180, and 200 °C treatments, respectively (Table 2). The effects of temperature, holding period and their interaction are similar to the mass loss. Oil heat treatment at 200 °C resulted to lower damage compared to the other temperature levels (*p* < 0.001). The effects of treatment duration from 30 to 120 min on the damage rating were not significant (*p* > 0.350). Interactions between temperature and holding period were also not significant (*p* > 0.747). Again, the results confirmed significant role of temperature in oil heat treatment.

It is important to note that termites started biting at the edges and moved towards the center of bamboo samples. They left behind only the fibers near the periphery and the rind portion after the 16-week exposure.

The present results somewhat differed from those obtained with Scots pine and Norway spruce [11]. In the latter, oil heat treatment alone is not enough to enhance resistance against subterranean termites. Also, impregnation with vegetable oil after oil heat treatment was needed for increased termite-resistance. Possibly, the oil heat treatment would not work in the same manner for wood and bamboo due to their anatomical differences. The oil in this study might have penetrated the bamboo samples, though no test was performed to verify the degree of retention. The type of oil might also have different effects [12]. The improved resistance could also be attributed to changes in the bamboo’s chemical composition during thermal modification such as degradation of the chemical components and/or the formation of toxic degradation products [13]. The exact reason for the thermally-modified bamboo’s enhanced resistance to subterranean termites necessitates further investigations.

**Table 2:** Summary of damage rating after a 16-week exposure of *D. asper* to *M. losbañosensis*.

Treatment Temperature (°C)	Holding Period (min)		
	30	60	120
140	99.00 ± 2.24 ^a^	95.00 ± 6.12 ^a^	83 ±13.96 ^ab^
160	94.00 ± 5.48 ^a^	75.00 ±27.39 ^ab^	82 ± 12.04 ^ab^
180	79.00 ± 12.94 ^ab^	79.00 ± 12.94 ^ab^	82 ± 18.91 ^ab^
200	57.00 ± 35.64 ^ab^	59.00 ± 27.70 ^ab^	46 ± 26.08 ^b^
control	100.00		

Means having the same letter/s in each column are not significantly different at α = 0.05.

## 4. Conclusions

The 16-week laboratory termite exposure test was done to determine the effects of oil heat treatment using virgin coconut oil. Improvement in resistance against subterranean termites varies with different temperature levels holding periods and their interactions. However, temperature has a more dominant effect on the termite resistance of oil heat treated *D. asper*. The exact causes of the improved resistance of thermally-modified bamboo need further investigations.
